# MARES, a replicable pipeline and curated reference database for marine eukaryote metabarcoding

**DOI:** 10.1038/s41597-020-0549-9

**Published:** 2020-07-03

**Authors:** Vanessa Arranz, William S. Pearman, J. David Aguirre, Libby Liggins

**Affiliations:** School of Natural and Computational Sciences, Massey University Auckland, Albany, Auckland, 0745 New Zealand

**Keywords:** Genetic databases, Biodiversity, Molecular ecology, Marine biology

## Abstract

The use of DNA metabarcoding to characterise the biodiversity of environmental and community samples has exploded in recent years. However, taxonomic inferences from these studies are contingent on the quality and completeness of the sequence reference database used to characterise sample species-composition. In response, studies often develop custom reference databases to improve species assignment. The disadvantage of this approach is that it limits the potential for database re-use, and the transferability of inferences across studies. Here, we present the MARine Eukaryote Species (MARES) reference database for use in marine metabarcoding studies, created using a transparent and reproducible pipeline. MARES includes all COI sequences available in GenBank and BOLD for marine taxa, unified into a single taxonomy. Our pipeline facilitates the curation of sequences, synonymization of taxonomic identifiers used by different repositories, and formatting these data for use in taxonomic assignment tools. Overall, MARES provides a benchmark COI reference database for marine eukaryotes, and a standardised pipeline for (re)producing reference databases enabling integration and fair comparison of marine DNA metabarcoding results.

## Abstract (summary)

**Table:** 

Measurement(s)	DNA
Technology Type(s)	bioinformatics analysis
Factor Type(s)	DNA sequence
Sample Characteristic - Organism	Eukaryota
Sample Characteristic - Environment	marine environment

Machine-accessible metadata file describing the reported data: 10.6084/m9.figshare.12324122

## Background & Summary

DNA metabarcoding has emerged as a powerful tool for quantifying biodiversity using genetic sequences^1,2^. Metabarcoding studies have helped us understand the biodiversity of difficult to sample environments, and to monitor important ecosystems^3^. Although some measures of species richness and diversity are attainable from the genetic data alone (e.g. based on unique sequence variants, or molecular operational taxonomic units), deriving species identities from the genetic sequence data is crucially important in many study contexts. Species assignment requires a reference database containing sequences that have been taxonomically assigned. However, the choice of database is known to affect the classification of sequences to species^4^ and constructing a curated reference database tailored to specific study objectives can dramatically increase the classification sensitivity, reduce false discovery rates, and prevent the overclassification of sequences to species^5^.

Constructing a custom reference database can be time consuming and requires specialist skills, and as a result, many researchers rely on pre-existing curated databases. Pre-existing databases are usually either: very taxonomically broad, with the goal of encompassing as many high-quality barcode sequences as possible; or are generated with a specific question in mind. Given the impact that the choice of reference databases can have on metabarcoding study inferences, there are numerous campaigns to compile publicly available barcode libraries for specific groups (e.g. photosynthetic eukaryotes, PhytoREF^6^; arthropods^5^; fungus, UNITE^7^) and geographic locations (e.g. aquatic life in European countries^8^, freshwater macroinvertebrates of Australia^9^). The use of such standardised reference databases for taxonomic assignment avoids possible biases in species determination introduced by the choice of reference database, thereby allowing unbiased comparisons among studies.

The marine realm is one of the richest and most diverse ecosystems on our planet, containing representatives from almost all the eukaryotic forms of life^10^. DNA metabarcoding in marine ecosystems has been used to assess environmental impacts^11^, undertake diet analysis^12^, understand trophic interactions^13^ and to track invasive species^14^. Common to all of these applications of marine metabarcoding, has been the creation and/or use of a reference database to assist in the taxonomic assignment of sequences^15^. Yet, to date, there has been no replicable creation or standardised use of a reference database for the taxonomic assignment and therefore biodiversity analyses of marine eukaryote diversity sampled using DNA metabarcoding.

The most commonly used marker for metazoans (a large component of marine eukaryotes) is the cytochrome oxidase 1 (COI) gene region which has been shown to successfully discriminate among species, and populations within species^16^. Large and rapidly growing COI barcode repositories provide an ideal resource for taxonomic identification and quantification of biodiversity^17^. For metazoan COI sequences, the Barcode of Life Database (BOLD)^18^ has become the preferred reference database due to in-built standards that ensure species identification^19^. Although GenBank^20^ is not curated to the same taxonomic standards as BOLD, its broader collection of eukaryotic sequences can increase accuracy by being informative at the genus- and species-level^21,22^. There is a considerable overlap between these two large repositories; however, there remain sequences and metadata that are unique to both. Therefore, the compilation of a reference database for COI sequences for marine eukaryotes would ideally draw from both GenBank and BOLD^17,23^ and would synonymize the species identities across sequences drawn from both repositories.

Here we present the MARine Eukaryote Species (MARES) database, providing reference sequences of the COI gene region for a large diversity of taxa found in marine ecosystems with standardised and curated taxonomic identifiers. The reference database has been built by combining all available sequences from GenBank and BOLD to increase the taxonomic coverage and confidence^34^. MARES includes only taxa from Eukaryote families that are represented in the marine environment, and is formatted for use in popular taxonomic assignment software (MEGAN^24^ and Kraken2^25^). The bioinformatic pipeline used to generate the MARES database is publicly available along with a tutorial to generate curated and comprehensive reference databases with normalised taxonomy, in a replicable manner. Using this bioinformatic pipeline, researchers will be able to choose which taxonomic groups are represented within their custom reference database and can incorporate the most recently published sequences. Importantly, the MARES pipeline enables users to participate in the decisions that need to be made in generating a sequence reference database that will have downstream consequences on their biodiversity inferences.

## Methods

**Fig. 1:**
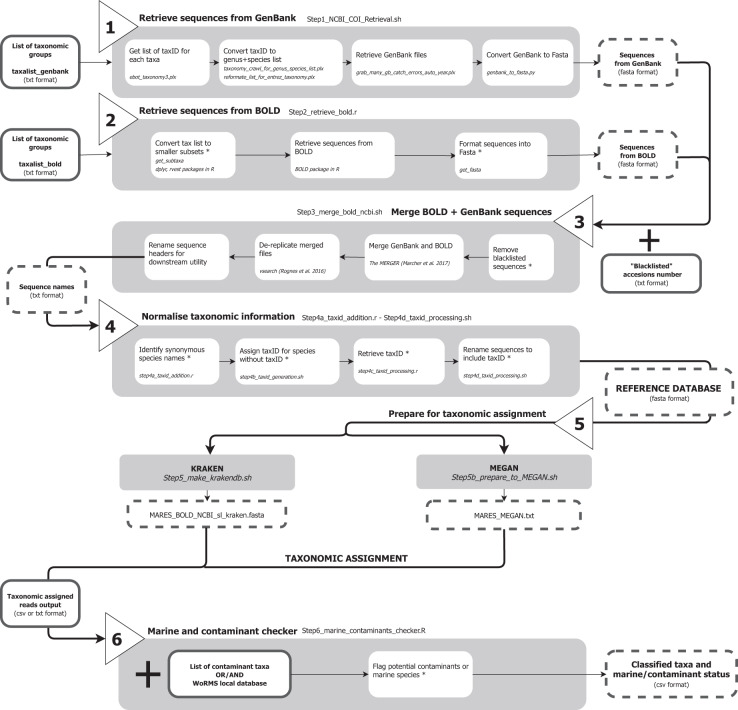
Bioinformatic pipeline for generating a custom reference database combining sequences retrieved from BOLD and GenBank for a taxonomic group of interest. Shaded boxes detail the workflow for each numbered step described in the methods and the name of the script required for each step (available on github: https://github.com/wpearman1996/MARES_database_pipeline). Smaller open boxes describe the subroutines including the functions, packages, and software required (in italics). Boxes with solid outlines indicate input files and boxes with dotted-lined boxes indicate the output files. Asterisks denote the original contributions of MARES to the previously published routines.

We present the MARES (MARine Eukaryote Species) reference database primarily as an example for how the pipeline can be used, and as a first step towards the standardization of reference databases and bioinformatics protocols to enable reliable comparisons among marine metabarcoding studies. The MARES reference database was generated using a combination of existing and custom-made scripts (Fig. 1). The bioinformatic pipeline, step-by-step tutorial, and input files relevant to the MARES database are available in the GitHub repository: https://github.com/wpearman1996/MARES_database_pipeline. The input files and arguments provided in the scripts are specific to the creation of a reference database for COI metabarcoding of marine eukaryotes. Nevertheless, these files and arguments can be easily modified. Below we provide the detailed outline of our pipeline, and how it was used to generate the MARES database.

### Taxa curation

To create a COI reference database for marine eukaryotic taxonomic assignment, we extracted a list of all families present in the World Register of Marine Species (WoRMS) database using a local copy provided by the WoRMS Editorial Board^26^(including contributions from AlgaeBase^27^). The number of sequences available for marine organisms in gene repositories is generally lower than for terrestrial organisms^16^. For this reason, we chose to include all species, including terrestrial species, within these families so that we retained the ability to assign the sequences to higher taxonomic levels (i.e. genus and/or family) when sequences for marine species were not available. As a further example use of our pipeline, we also created a COI reference database for a list of taxa that are common contaminants (e.g. *Homo sapiens*) to assist with detection of contaminant sequences as an optional quality control step in metabarcoding studies. Both the list of marine-relevant families and the common contaminants list can be modified according to user preferences when using the MARES pipeline, as described in the following steps (also see Fig. 1).

#### GenBank sequence retrieval

Sequences were retrieved from GenBank using modified scripts from Porter and Hajibabaei^17^. These scripts require the list of focal taxa as a text file (in our case, this was the list of marine-relevant families and the common contaminants list). The list of focal taxa is then converted to a list of TaxIDs, and the TaxIDs for all subtaxa are retrieved and converted to binomial names. These binomial names were used as search terms alongside the gene region name (and synonyms, i.e. Cytochrome Oxidase 1 has synonym names of CO1, COI, COX1 and COXI), upload data (2003 to 2019), origin (Eukaryota) and searches with and without “barcode” as a keyword (MARES_COI_BAR and MARES_COI_NOBAR, respectively). Additional search parameters can be defined by the user in the script *grab_many_gb_catch_errors_auto_CO1_year.plx*, such as the sequence length, sequence source (mitochondrial or nuclear) or including keywords. The GenBank flat files are then retrieved and stored locally and converted to fasta format, using the *genbank_to_fasta.py* script.

#### BOLD sequence retrieval

Using the *bold* package^28^ in R^29^ and the functions provided in the *step_2_retrieve_bold.r* script (Fig. 1), sequences for all taxa provided in our focal taxa list were retrieved, and formatted as a fasta file. Additionally, because the number of sequences that can be retrieved at any one time is limited by the amount of available RAM, we provide functions to retrieve a list of immediate subtaxa to enable staged retrieval.

#### Merge GenBank and BOLD sequence files

An optional initial step, prior to merging, is to exclude specific accession numbers, that for instance, are sequences where the taxa are suspected of being misidentified. This is done by adding accession numbers to the blacklisted_accessions.txt file. Sequences containing these accession numbers within the headers are then removed prior to merging the two databases. In order to combine the GenBank and BOLD fasta files of retrieved sequences, we used scripts modified from Macher, *et al*.^23^. The merged file was then dereplicated using *Vsearch*^30^, to remove any sequences that were duplicated, leaving only unique sequences. Last, the headers for these files are modified to enable use in MEGAN.

#### Taxonomic information normalisation (Quality control)

The “Lowest Common Ancestor” (LCA) assignment algorithm is widely used for taxonomic classification. The LCA approach requires a taxonomy tree to assign the read to the lowest common ancestor if the read is not initially assigned to a single taxon. The NCBI Taxonomy is used as the standard nomenclature and classification repository for the International Nucleotide Sequence Database Collaboration (INSDC), comprising the GenBank database^31^. However, BOLD uses a slightly different taxonomy. In this step, we normalise the taxonomic information of the taxa names included in our combined sequence database by attaching a NCBI taxonomy identifier (hereafter TaxID) to each taxon name. The addition of these TaxIDs to taxa names is beneficial for comparisons among studies and standardisation of methods^32^. Moreover, the addition of TaxIDs to all reference sequences is crucial for taxonomic assignment algorithms, such as those implemented in MEGAN, Kraken2 or *ecotag*^33^.

To confirm each sequence had a TaxID, the list of species represented in the merged fasta file was processed in the GenBank TaxIdentifier program, using the *step4a_taxid_addition.r* (Fig. 1). This tool retrieves TaxIDs for all species in the merged fasta file and identifies any other appropriate synonyms to ensure that synonymous species retain the correct TaxID. If a taxon in the database does not have a TaxID but the genus can be identified, then a unique identifier is created and appended to the local NCBI taxonomy^34^. The new TaxID is assigned to the species level, nested within the appropriate genus and higher taxonomic ranks. New TaxIDs are generated by identifying, and adding to, the largest TaxID present in the local NCBI taxonomy, as such the specific TaxID created will vary among versions of the taxonomy. Note that this solution is only suitable for taxa where genus information can be obtained (removing this limitation is a possible future development of the MARES pipeline). Last, if a TaxID cannot be found or generated by the methods described above, then the sequence is removed from the database.

Following this process, a new fasta file is generated, now including information regarding the gene region name, the species or genus names, the relevant accession numbers for the sequence, as well as the TaxID. Owing to the keyword search approach used (see above), the length and position of the retrieved reference sequences for the COI gene region may be highly variable. Accordingly, users may opt to include an additional step at this point, such as sequence alignment and/or *in silico* PCR, in order to refine the database to certain target regions (such as the Leray fragment^35^ or I3-M11 partition^36^). Alternatively, depending on the high-throughput sequencing platform, the user may instead prefer to retain longer reference sequences suitable for classifying longer sequence reads^37^.

#### Preparation for taxonomic assignment using MEGAN and Kraken2

MEGAN and Kraken2 are commonly used software for taxonomic assignment. MEGAN uses an alignment-based method for classification. It maps long reads against a reference database and uses the LCA approach to assign the reads in the phylogeny. Kraken2 examines the k-mers within sequences and uses the k-mers to query the taxonomic information from a reference database also using the LCA approach to map them. We used *step5_makekrakendb.sh* and *step5b_prepare_to_MEGAN.sh* (Fig. 1) to prepare the fasta files for use in both MEGAN and Kraken2. The first script in this step prepares a fasta file for Kraken2, with the syntax “kraken:taxid|TaxID”, and then this is converted into a Kraken2 database within the Kraken2 software package. The second script generates a BLAST database from the normalized fasta file, using the makeblastdb function within BLAST.

#### Check marine taxa and/or contaminants in the reference database

The user might be interested in distinguishing marine taxa and/or identifying common contaminants in the generated database. The pipeline offers an additional *step6_marine_contaminants_checker.R* which can be used to specifically identify which taxa in the reference database are marine based on the WoRMS local database. Alternatively, this script can help flag potential contaminants, based on a user-defined list of contaminants. The user can then decide whether to remove contaminant sequences from the sequence reference database or merge the contaminant sequences into the reference sequence databases.

## Data Records

MARES_COI_BAR and MARES_COI_NOBAR^38^ are available on the Open Science Framework (10.17605/OSF.IO/8RDQK) in formats suitable for MEGAN or Kraken2. The total number of sequences and number of unique sequences in the MARES databases, relative to other existing COI reference databases, are detailed in Table 1. The pipeline and tutorial for generating the databases are available in the GitHub repository: https://github.com/wpearman1996/MARES_database_pipeline.

**Table 1:** Published reference databases commonly used for taxonomic assignment in COI eukaryotic metabarcoding studies.

Reference Database	Target organisms	Source repository	Method	Sequences	Unique species (%Unique species)	Marine species (%Marine species)	Reference
BOLD	Eukaryotes	BOLD	Keyword search	5,586,934	169,705 (3.04)	18,328 (10.80)	Ratnasingham and Hebert^18^
GenBank	Eukaryotes	GenBank	Keyword search	1,933,547	160,061 (8.28)	17,943 (11.21)	NCBI Resource Coordinators^20^.
Midori	Metazoans	GenBank	Keyword search	927.386	131,988 (14.23)	14,057 (10.65)	Machida, *et al*.^43^
db_COI_MBPK	Eukaryotes	EMBL, BOLD	*in silico* ecoPCR + custom R script	188.975	48,853 (25.85)	6,844 (14.01)	Wangensteen and Turon^39^
CRUX_CO1	Eukaryotes	EMBL, GenBank	CRUX (*in silico* ecoPCR + blast)	1,401,802	127,422 (9.10)	15,737 (12.35)	Curd, *et al*.^41^
MARES_BAR	Marine eukaryotes	GenBank, BOLD	Keyword search	1,224,187	61,123 (4.91)	17,884 (29.26)	This data descriptor^38^
MARES_NOBAR	Marine eukaryotes	GenBank, BOLD	Keyword search	1,491,691	71,499 (4.79)	19,154 (26.79)	This data descriptor^38^

BOLD and GenBank reference databases were built using Step 1 and 2 of the bioinformatic pipeline (Fig. 1). ‘BOLD’ was generated by retrieving all COI sequences available from the BOLD repository. ‘GenBank’ was generated with the keyword search Eukaryota and COI synonyms. ‘Unique species’ were retained after a quality control procedure that retains only unique, fully identified taxa with binomial species names. ‘% Unique species’ was calculated using the number of unique species as the numerator and the total number of sequences as the denominator. ‘Marine species’ was determined by the number of unique species present in each database that appeared in the World Register of Marine Species (WoRMS) and AlgaeBase^27^. ‘% Marine species’ was then calculated using the number of marine species as the numerator and the number of unique sequences as the denominator.

## Technical Validation

To highlight the value and potential utility of our curated reference databases (MARES_COI_BAR and MARES_COI_NOBAR) we compared them with previously published reference databases for the metabarcoding locus COI (Table 1). The published reference databases varied considerably in the number of taxa represented, likely owing to the date of sequence retrieval (i.e. sequence repositories continue to grow in taxonomic breadth and number of sequences), the ways in which they were created (e.g. source repositories and retrieval approach), and how they were curated in preparation for their intended use-case. For instance, current approaches to generate databases can include the use of *in silico* PCR, mixed approaches such as those used to generate db_COI_MBPK^39^ or the CRUX approach^40^ used in CRUX_CO1^41^, which result in larger locus specific reference databases^40^ overcoming the problems raised by merely using *in silico* PCR where relevant sequences without the primer regions can be omitted^42^. Keyword searches in public sequence repositories such as BOLD, GenBank and Midori^43,44^ are also commonly used, but these can be limited by metadata accuracy. Although sequence references databases truncated to a target amplicon length facilitate species-level assignment when they are designed for specific groups of taxa (e.g. in marine nematodes^45^), it is unlikely that this better performance extends to cases where the focal taxa may be from many different phyla, and therefore the amplicon lengths, and/or which primers sets were successful is also unknown. An important step in generating the MARES databases was the curation for all marine taxonomic groups. Although our curation procedure reduced the total number of unique species, the MARES databases have more marine species, and the proportion of marine species is between two and three times higher, than the other reference databases (Table 1). As a result, MARES is useful for marine metabarcoding studies covering a wide taxonomic range and avoids the computational burden of having an unnecessarily large sequence reference database.

To compare the MARES databases with other published COI reference databases in terms of taxonomic composition, we used pairwise beta (β)‐diversity^46^ measures based on the presence and absence of taxa within each database. Total Jaccard’s dissimilarity β_JAC_ can be partitioned into two components: the β_JNE_ nestedness-resultant component which indicates the elimination or addition of species in one of the two compared databases (i.e. a change in richness); and the β_JTU_ turnover component which indicates the substitution of a species in one database for a different species^47^. Species presence-absence matrices were generated for each database (Scripts available on github: https://github.com/wpearman1996/MARES_database_pipeline) and three pairwise dissimilarity matrices describing the total dissimilarity, the nestedness-resultant component, and the turnover component among datasets were calculated using the beta.pair function of the package ‘betapart ver. 1.5’ in R^48^. The ratio of the nestedness-resultant (β_JNE_) and total Jaccard’s dissimilarity (β_JAC_), hereafter β_ratio_, was calculated to show the proportion of dissimilarity explained by each of the β_JAC_-diversity components. A β_ratio_ less than 0.5 would indicate a stronger contribution of the turnover component whereas a large value for the ratio greater than 0.5 would indicate a stronger contribution of the nestedness-resultant component indicative of differences driven by database size rather than turnover of species identities.

Of the databases we compared, BOLD and GenBank contained the greatest number of species, likely due to the fact that they are the main sequence resources, followed by CRUX_CO1 and Midori (Table 2). The MARES databases and the db_COI_MBPK database were similar in the number of species retained (Table 1), but notably different in species composition (Table 2; β_JAC = _0.80 and 0.82 for MARES_COI_BAR and MARES_COI_NOBAR, respectively). Furthermore, differences between db_COI_MBPK and the MARES databases were driven almost entirely by the turnover of species identities (Table 2; β_ratio_ = 0.04 and 0.06 for MARES_COI_BAR and MARES_COI_NOBAR, respectively). Although the large turnover in species identities between MARES and db_COI_MBPK was somewhat surprising, this result underscores the importance of the choice of sequence repository (e.g. EMBL vs. BOLD) and methodological differences (mixed approaches vs. keyword searches) in the construction of a reference database. These databases were also generated five years apart and the availability of genetic data has increased dramatically over this time, highlighting the importance of updating the databases periodically given the exponential increase in the size of these repositories. The Midori and CRUX_CO1 databases contained between 2.4 and 2.8 times more species than the MARES databases, and differences in species composition between the MARES databases and Midori as well as CRUX_CO1 were large (Table 2) and driven by a combination of turnover and nestedness (Table 2). Comparing BOLD and GenBank, we found a Jaccard dissimilarity of 0.34 (Table 2), and it was the turnover component that contributed most strongly to the dissimilarity between BOLD and GenBank (Table 2; β_ratio_ = 0.10). This result affirms the value in drawing from both the BOLD and GenBank databases in order to create the most comprehensive reference database. In contrast, when all other databases were compared to GenBank and BOLD, the differences in species composition were primarily due to nestedness (i.e. richness differences), as expected (Table 2). The smallest difference among databases was found between our two curated databases (β_JAC = _0.15), and this difference was solely due to the greater richness of species in the MARES_COI_NOBAR database compared with MARES_COI_BAR (β_ratio_ = 0.92). The difference between MARES_COI_BAR and MARES_COI_NOBAR was the addition of the Keyword “barcode” to each query from GenBank in MARES_COI_BAR to retrieve only high quality records^49^ thereby reducing the number of sequences and species included. We leave the decision as to which MARES database is most appropriate for each use-case to the user, depending on their specific purposes.

**Table 2:** Pairwise β‐diversity measures for comparisons of species composition among reference databases Below the diagonal is the total Jaccard’s dissimilarity (β_JAC_) and above the diagonal is the β_ratio_ representing the proportion of total Jaccard’s dissimilarity (β_JAC_) explained by nestedness (β_JNE_).

	Midori	BOLD	GenBank	db_COI_MBPK	MARES_COI_ NOBAR	MARES_ COI_BAR	CRUX_CO1
Midori		0.32	0.57	0.64	0.26	0.32	0.11
BOLD	0.43		0.10	0.87	0.53	0.81	0.43
GenBank	0.26	0.34		0.81	0.62	0.65	0.59
db_COI_MBPK	0.70	0.73	0.72		0.06	0.04	0.82
MARES_COI_NOBAR	0.70	0.68	0.64	0.82		0.92	0.27
MARES_COI_BAR	0.73	0.67	0.69	0.80	0.15		0.39
CRUX_CO1	0.23	0.40	0.29	0.65	0.69	0.69	

Smaller values for the ratio indicate that dissimilarities are primarily due to databases containing different species, whereas larger values indicate dissimilarities are primarily driven by differences in the number of species present in each database.

Here we present the MARES reference database in compatible formats for two common taxonomic assignment software providing a standard reference database for the burgeoning array of marine metabarcoding studies. The MARES_BAR and MARES_NOBAR databases include 61,123 and 71,499 unique COI sequences, with representatives of from 2,638 and 2,841, respectively for MARES_BAR and MARES_NOBAR, of the 5,500 families known to comprise marine taxa^26,27^. Although many of the sequences retrieved for these families are from terrestrial relatives of marine taxa, these sequences allow us to at least classify these sequences to genus and/or family levels. Our unique curation approach results in the MARES databases having a higher proportion of marine species than other available reference databases (Table 1). It is important to note however, that although our databases have the greatest marine representation for a reference database, the proportion of all marine taxa represented is still low highlighting the need for more research and funding to sequence marine taxa. Furthermore, we provide a replicable pipeline outlining the steps required to reproduce or update the MARES databases, or to produce a reference database similarly suited to other taxonomic groups. Our pipeline and tutorial are designed to help molecular ecologists who are unfamiliar with the important choices required when using, or creating, a reference sequence database for metabarcoding. MARES enable users to determine the sequence repository design and provides downstream analytical tools to quantify the consequences of design decisions on database composition.

## Usage Notes

- A detailed tutorial is provided on the GitHub repository for how to replicate the MARES databases or to implement these steps to generate a user-defined reference database for different taxa and gene regions.
- An NCBI API key is required for the technical validation that uses the *taxize* R package^50^.
- Your email address must be inserted on line 86 of *ebot_taxonomy3.plx*, and line 32 of *grab_many_gb_catch_errors_auto_CO1_year.plx*
- The default search terms can be modified on line 29 of *grab_many_gb_catch_errors_auto_CO1_year.plx*
- This script requires a list of taxa that should be included in the database. The input files for replicating the MARES databases are available on the GitHub repository: https://github.com/wpearman1996/MARES_database_pipeline
- The taxa list can be at any taxonomic level, and all subtaxa will be retrieved. For example, if you wish to download all Chordata, but exclude Actinopterygii, then you must download all subtaxa within Chordata at the same taxonomic level as Actinopterygii, rather than download all of Chordata.
- You must specify the location of the NCBI taxonomy nodes.dmp and names.dmp files on lines 26 and 27 of *taxonomy_crawl_for_genus_species_list.plx*
- We also provide scripts to generate custom Kraken2 databases, however as with the rest of the scripts, it is necessary to have a local copy of the NCBI taxonomy files.

## Data Availability

Scripts used to generate the MARES reference databases and technical validation are freely available from https://github.com/wpearman1996/MARES_database_pipeline
